# Diagnostics, incidence and treatment of the distal radius fractures: an area for many studies, opinions and treatment options

**DOI:** 10.1007/s00068-022-02150-3

**Published:** 2022-11-30

**Authors:** Johannes Frank

**Affiliations:** Department of Trauma, Hand and Reconstructive Surgery, University Hospital Goethe-University Frankfurt, Theodor-Stern-Kai 7, 60590 Frankfurt, Germany

Conservative treatment of distal radius fractures could be found in historical articles for more than 3000 years including techniques for reposition and splinting. Since surgical treatment has been introduced by Alan Lambotte around 1908, surgical treatment of distal radius fractures has certainly increased in recent decades, especially since the introduction of new angular stable implants around 30 years ago [1, 2]. However, many studies, metanalysis and systematic reviews are available in the literature, discussing all facets of this fractures.

Due to the high incidence, around 400–700 per 100.000 persons, first of all, epidemiological and diagnostical studies are worthwhile to be addressed. These are dealing with the problems in emergency departments to establish an effective diagnostic algorithm not missing a significant amount of fractures and also not to over-diagnose considering the given resources [3, 4].

Second, the treatment modalities are in focus of various publications to provide guidelines not to over-operate. Such recommendations are available in various countries around the world, e.g. guidelines of the German Orthopaedics and Trauma Surgery Society (DGOU) or the British Orthopaedic Association (BOA) and British Society for Surgery of the Hand (BSSH). These are validated with respect to the indication for surgical therapy according to the given “thresholds for intervention” like summarised in such publications [5–9]. Following such measures, some authors conclude for themselves, that surgical therapy was chosen too often [5].

Certainly, an extensive literature review could be sought here [10]. Guidelines as noted earlier may be helpful and timesaving. However, the limits of conservative treatment also known from “historical literature” can be summarised as follows:

- Dorsal tilt of 20° – 67% leads to osteoarthritis development [11].
- Up to 5 mm relative ulnar advancement is tolerable with good outcome [12].
- Starting at 2 mm relative ulnar advancement results in measurable functional deficits [13, 14].
- Joint incongruity > 1 mm might result in more than 90% of the patients in osteoarthritis [12].
- Development of mediocarpal problems after malunion occurs within weeks to months [15].

Most listed measurement values are typical for studies in this area; however, there is a need to include the anatomical variances [16]:

- Ulnar inclination 22° (13–30°)
- Palmary inclination 12° (0–28°)
- Radial length 12 mm (8–18 mm)
- Ulnar variance ± 0 mm (− 4 to + 4 mm)

The discussion continues, if an adequate conservative therapy is not possible, about the various options for stabilisation and/or postoperative care [10, 17, 18]. Publications all around the world should be appreciated since some expensive implants are not everywhere available. Surgical treatments and studies published still include all known surgical options:

- External fixation
- K-wire stabilisation
- Screw fixation
- Nails
- Plates
- The combination of implants

The various surgical techniques can be further subdivided, mostly according to the fracture pattern, using an appropriate classification to be able to compare the data. The influence of concomitate injuries (ligaments, ulna, etc.) is much more difficult to adjust in studies dealing with complex fractures [10, 17, 18].

The reader is exposed to numerous studies which are available on distal radius fracture and therefore, a pragmatism for the treatment algorithm at each centre is necessary to define a strategy and establish a framework for decisions for conservative or surgical care. Particularly this issue focuses on this topic, as well as in almost every issue of the European Journal of Trauma and Emergency Surgery, publications around the distal radius can be found.

The final therapy must be discussed individually with the patient. The specific demands, needs and health status must be weighed against the extent of the fracture and possible complications. A discussion must consider that conservative therapy also requires appropriate experience (anaesthesia, reduction technique, immobilisation) and can vary greatly depending on the centre or even the country. As a result, complications also arise not only from operative treatment, but as well from conservative therapy and require a scientific analysis (e.g., CRPS). However, looking at the publications dealing with surgical interventions, one can assume that the knowledge of conservative therapy has deteriorated in recent years, at least in countries exposed to all surgical possibilities.

Nevertheless, age grouping is also very imported, as well as underlying diseases and bone quality [19, 20]. For some surgeons, a surprisingly large population is still treated conservatively with success. Considering age-adjusted therapy for such fractures, it will always be a challenge to perform clinical studies [21–24]. Good results with conservative therapy dislocated radial fractures at age > 60 years of age have been published [20]

An optimal study might be a selection of a defined fracture type, homogeneous group size and no statistical differences in health profile. It is obvious that a comparison of conservative therapy would be necessary, with recording of the complications of both groups (including procedure changes, soft tissue problems, CRPS, also the number of conservative interventions, revisions in surgical therapy). The final outcome would have to be measured (radiological outcome, functional values with wrist score) and quality of life (e.g., DASH score) evaluated. Regarding the results, a final prognosis assessment should be performed [25].

Summarising the discussion about the treatment of distal radius fractures, a final statement might be the intent to perform good prospective randomised trials.
